# Hepatitis B virus reactivation after successful treatment of hepatitis C virus with sofosbuvir and ribavirin: A case report and literature review

**DOI:** 10.1097/MD.0000000000022650

**Published:** 2020-10-09

**Authors:** Akio Miyasaka, Yuichi Yoshida, Akiko Suzuki, Tomoyuki Masuda, Hiroaki Okamoto, Yasuhiro Takikawa

**Affiliations:** a Division of Hepatology, Department of Internal Medicine; b Department of Pathology, Iwate Medical University School of Medicine, Yahaba-cho, Shiwa-gun, Iwate; c Division of Virology, Department of Infection and Immunity, Jichi Medical University School of Medicine, Shimozuke, Tochigi, Japan.

**Keywords:** hepatitis B reactivation, chronic hepatitis C, direct-acting antiviral agents

## Abstract

**Rationale:**

Hepatitis B virus (HBV) reactivation caused by immunosuppressive therapy or chemotherapy is well known. The administration of direct-acting antiviral agents (DAAs) to treat hepatitis C virus (HCV) infection has also been reported to cause HBV reactivation. We report a rare case of HBV reactivation in a patient with HCV infection after DAA therapy.

**Patient concerns:**

In 1996, a 53-year-old female was identified as infected with HCV at a medical check-up, following which she visited our hospital. She was infected with HCV genotype 2b, and at follow up in 1997, was found to be hepatitis B surface antigen (HBsAg) and antibody against HBsAg negative, antibody against HBV core positive. She then experienced malignant lymphoma in 2001 at 58 years of age. Complete remission was achieved following chemotherapy and autologous peripheral blood stem cell transplantation. In 2014, she remained negative for HBsAg and antibody against HBsAg but positive for antibody against HBV core. In 2015, 12 weeks of sofosbuvir and ribavirin treatment for HCV was started. Serum HCV RNA levels rapidly decreased, and HCV elimination was confirmed at 24 weeks after cessation of DAA treatment. Acute hepatitis B developed at 15 weeks post- sustained virological response without any symptoms and physical examination findings.

**Diagnoses:**

This case is speculated to represent HBV reactivation induced by DAA treatment in a patient with previously resolved HBV, based on virologic and clinical status. Genome sequencing revealed the HBV genotype as A2.

**Interventions:**

The patient was treated with nucleotide analog for HBV reactivation once a day.

**Outcomes:**

Serum HBV-DNA levels decreased, and serum liver enzymes improved following initiation of nucleotide analog treatment. Also, adverse events of nucleotide analog treatment were not observed.

**Lessons:**

Although the risk may be very low, DAA therapy can cause HBV reactivation in chronic hepatitis C patients with prior HBV infection. Thus, those patients must be closely monitored for serum HBV DNA levels during and after DAA treatment.

## 1 Introduction

Hepatitis B virus (HBV) infection shows a variety of clinical courses, including reactivation of previously resolved infection. Such HBV reactivation caused by immunosuppressive therapy or chemotherapy is well known. Risk of reactivation depends on the HBV infection status (chronic, inactive, and resolved hepatitis) and the immunosuppression status of the patient. Furthermore, reactivation may develop not only during immunosuppressive therapy or chemotherapy but also after its interruption or cessation. Moreover, HBV reactivation in chronic hepatitis C patients who have received direct-acting antiviral (DAA) therapy has been known.^[1,2]^

We herein describe a case of HBV DNA reappearance in a patient with resolved HBV infection-that is, in a patient with negative results for hepatitis B surface antigen (HBsAg) and the corresponding antibody (anti-HBs) and positive results for antibody against HBV core (anti-HBc)-who received anti-hepatitis C virus (HCV) treatment with sofosbuvir (SOF) and ribavirin (RBV).

## 2 Case report

**Figure 1:**
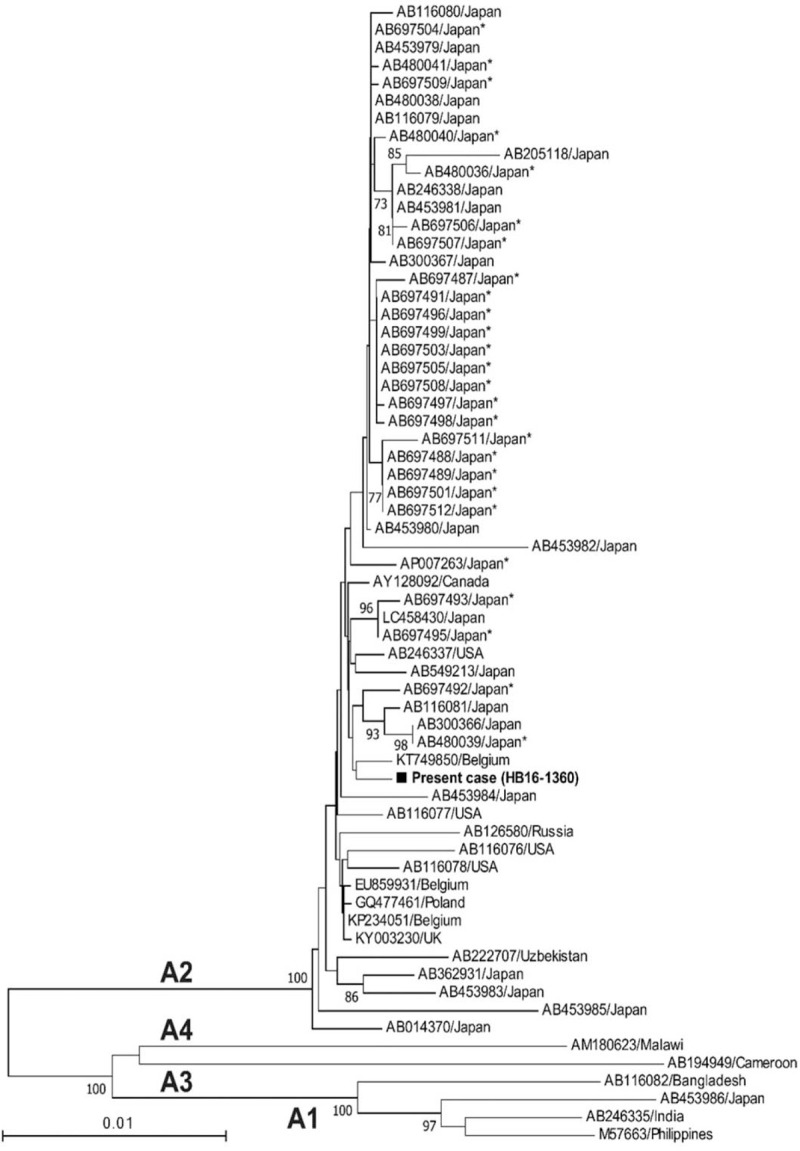
Phylogenetic tree based on the complete genomic sequences of genotype A hepatitis B virus (HBV) strains. A phylogenetic tree was constructed according to the neighbor-joining method based on the entire genomic sequences of the HBV strain (HB16-1360) obtained from the present case and representative genotype A HBV strains of subgenotypes A1 to A4. The HB16-1360 strain is highlighted in bold type with a closed box for visual clarity and the reported HBV strains are indicated with accession number and the name of the country where the strain was isolated. Asterisks indicate the HBV strains recovered from patients co-infected with human immunodeficiency virus or those who contracted acute HBV infection via sexual contact. Bootstrap values of > 70% are indicated for the nodes as a percentage obtained from 1000 resampling analyses of the data. The scale bar is in units of nucleotide substitutions per site.

**Figure 2:**
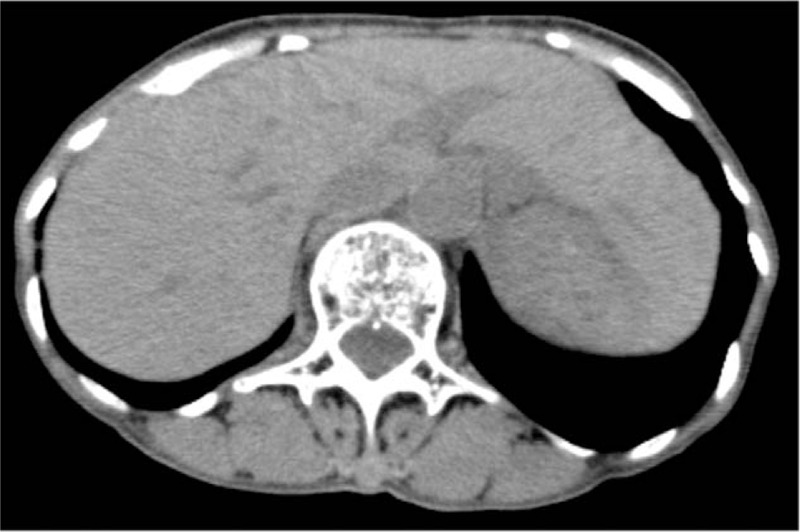
Imaging study of the abdominal computed tomography at hepatitis B virus reactivation.

**Figure 3:**
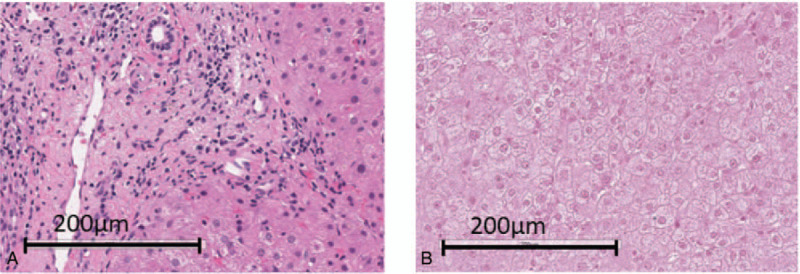
Histological findings of liver biopsy specimens. Liver biopsy showed (A) slight lymphocyte infiltration in the portal tract without obvious necrosis in the parenchyma (hematoxylin and eosin staining) and (B) no positive Victoria blue staining in hepatocytes (Victoria blue staining).

**Figure 4:**
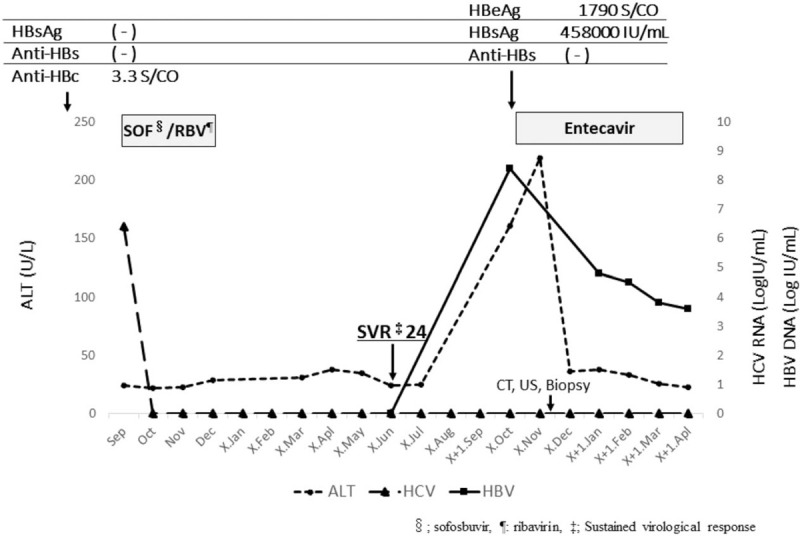
Clinical course. Hepatitis B surface antigen (HBsAg) and antibody against HBsAg were negative, antibody against hepatitis B virus (HBV) core was positive before direct-acting antiviral treatment. Treatment with sofosbuvir (SOF) + ribavirin (RBV) promptly decreased serum hepatitis C virus RNA levels, thereafter hepatitis C virus RNA remained undetectable. Serum alanine transaminase suddenly increased after treatment with SOF + RBV. Simultaneously, HBsAg became positive, while anti-HBs remained negative, and serum HBV DNA level increased, reaching 8.9 LogIU/mL at 39 weeks post cessation of direct-acting antiviral treatment with SOF + RBV. Entecavir was then administered. Liver function improved upon initiation of entecavir treatment. Moreover, serum HBV-DNA level gradually decreased.

A Japanese woman with a history of gastroduodenal ulcer was identified as infected with HCV at a medical check-up in 1996, when she was 53 years of age. She then visited our hospital. At this time, aspartate aminotransferase and alanine aminotransferase levels were 36 U/L, and 40 U/L, respectively. She has been followed up since then. As of 1997, HBsAg and anti-HBs were negative, but anti-HBc was positive, and she had no obvious family history of HBV. In 2001, she experienced malignant lymphoma (B-cell lymphoma) at 58 years of age. She received chemotherapy (Pirarubicin hydrochloride, Cyclosporine, Oncovin, Prednisolone), and then autologous peripheral blood stem cell transplantation was performed the next year, thereby, she achieved complete remission. In 2014, HBsAg and anti-HBs were negative, while anti-HBc remained to be positive. The patient then decided to seek treatment for her HCV infection. Genotyping revealed HCV genotype 2b and HCV RNA level was 6.4 LogIU/mL. Combination DAA treatment with SOF at 400 mg/d and RBV at 600 mg/d commenced in October 2015. HCV RNA was negative at 4 weeks post-initiation of SOF/RBV, and HCV RNA levels remained undetectable thereafter. This treatment continued for 12 weeks without severe adverse events. Sustained virological response was achieved at 24 weeks after cessation of this treatment. Moreover, at this time, aminotransferase levels were within the normal range, and serum HBV DNA level was negative. Thirty-nine weeks post DAA cessation, liver enzymes were quite suddenly elevated (aspartate aminotransferase 183 U/L, alanine transaminase 161 U/L) (Table 1) without any symptoms and physical examination findings. However, international normalized ratio of prothrombin time was not prolonged. HBsAg became positive and serum HBV-DNA level increased to more than 9.1 LogIU/mL. The entire genomic sequence of the HBV strain (HB16-1360) obtained from this patient was determined according to the previously described method^[3]^ deposited in the DDBJ/EMBL/GenBank databases (LC517161). The HBV strain was typed as genotype A (subgenotype A2) and harbored no mutations leading to HBeAg-negative phenotype such as G1896A and A1762T/G1764A. A phylogenetic tree based on the full-length genomic sequence revealed that the HB16-360 strain obtained from the present case shared the highest similarity (99.7%) with the Belgian strain (KT749850) and did not form a cluster with the HBV strains obtained from patients co-infected with human immunodeficiency virus or acute hepatitis patients who acquired HBV infection via sexual contact (Fig. 1). Abdominal computed tomography scan and ultrasound sonography revealed chronic changes in the liver without liver atrophy or ascites (Fig. 2). The patient received entecavir for suspected acute hepatitis caused by HBV reactivation to prevent progression to severe liver failure. In this case, a liver biopsy was performed at 43 weeks after the cessation of the treatment with SOF/RBV, and histological examination of liver biopsy specimens revealed no findings suggestive of self-limited acute hepatitis or positive Victoria blue staining in hepatocytes (Fig. 3). Liver function improved upon initiation of entecavir treatment. Moreover, serum HBV-DNA levels showed a gradual decrease to 3.6 LogIU/mL after 6 months of entecavir treatment (Fig. 4). Adverse events of nucleotide analog therapy were not observed. Thus, entecavir treatment successfully controlled HBV replication.

**Table 1:**
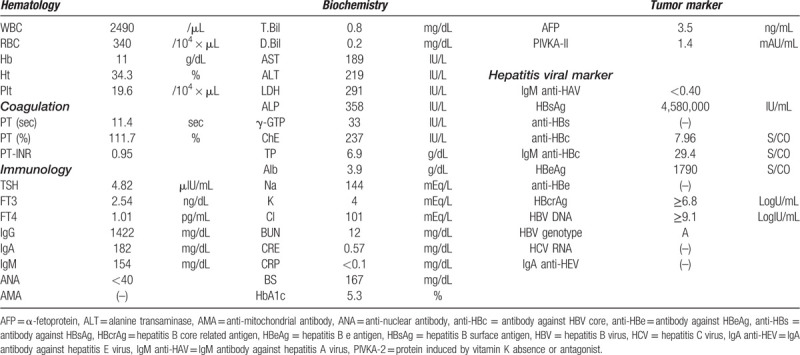
Laboratory data at 39 weeks after the cessation of treatment with sofosbuvir + ribavirin.

Written informed written consent was obtained from the patient for publication of this case report and accompanying images. Ethics approval was not required by our institution for completion and publication of this case report.

## 3 Discussion

HBV reactivation has been observed in inactive carriers (HBsAg-positive) or patients with resolved infection (HBsAg-negative and anti-HBc- and/or anti-HBs-positive) who have undergone immunosuppressive therapy and/or chemotherapy. DAAs for chronic HCV infection have been reported to induce HBV reactivation. We summarize previous case reports on HBV reactivation and/or HBV DNA reappearance during DAAs treatment in Table 2. According to those case reports,^[4–10]^ patient HBV infection status was varied including inactive HBV carriers, occult hepatitis B, and resolved infection. The time between initiation of DAAs and HBV reactivation also differed between cases.

**Table 2:**
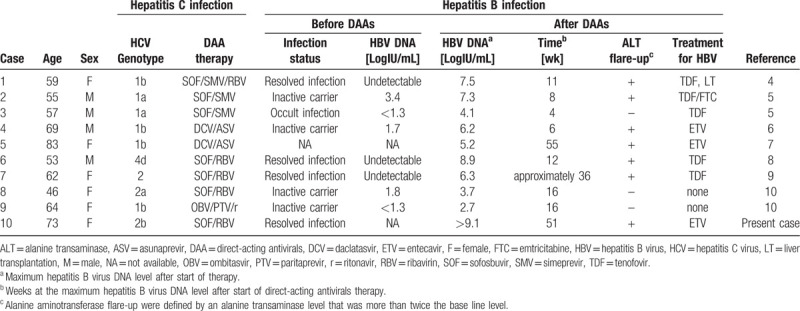
Case reports on hepatitis B virus reactivation among hepatitis B virus/hepatitis C virus C co-infected patients treated with direct-acting antivirals.

Regarding occurrence of HBV reactivation and/or HBV DNA reappearance during DAA treatment in Japan, there were several reports showing that clinical HBV reactivation and HBV DNA reappearance in HBsAg-positive patients treated with DAAs ranged from 25% to 50%.^[11–13]^ However, HBV reactivation and/or HBV DNA reappearance in HBAg-negative, anti-HBc- and/or anti-HBs-positive patients treated with DAAs was rare (range, 0.1%–2.6%).^[11–14]^ In this case, anti-HBc was positive, and HBsAg and anti-HBs were measured before and after DAA treatment, showing negativity before treatment but becoming positive at 39 weeks post treatment cessation in the case of HBsAg. Therefore, we speculated this case could be defined as HBV reactivation, with the following features suggestive of this scenario. Acute hepatitis mainly arises in young individuals because the route of transmission depends on sexual activity. This case involved an elderly woman who might be considered to be at low risk for this transmission route. She had no family history of HBV, and no opportunity for occurrence of infection was evidenced. Moreover, the HBV subgenotype was A2. There was a report from Japan that almost all strains from homosexual men belonged to subgenotype A2 following phylogenetic analysis of genotype.^[15]^ However, the current strain was different to those recognized in Japan. Furthermore, a histological examination of liver biopsy specimens revealed that there were no findings suggesting acute hepatitis. HBV spontaneous reactivation in elderly patients ^[16]^ unrelated to DAA treatment cannot entirely be ruled out, however we consider that it is unlikely.

The mechanism of HBV reactivation and HBV DNA reappearance occurring during DAA treatment is not fully understood. The following mechanism, however, has been proposed:

(i) HBV replication may be suppressed by HCV infection^[17–20]^;
(ii) HCV replication can be inhibited by an HBV superinfection^[21]^;
(iii) phase dominance of 1 virus over the other may alternate^[17,20]^;
(iv) the host immune response to both HCV and HBV may be attenuated by the eradication of HCV and thereby cause HBV reactivation.^[22,23]^

In the present case, absence of HBV reactivation during the previous chemotherapy indicates that HBV replication may be strongly suppressed by HCV infection. However, eradication of HCV by DAA treatment without inhibitory effects on HBV would be able to trigger reactivation of HBV infection. In addition, the host immune response may be attenuated by the eradication of HCV.

Japan Society of Hepatology guidelines state that the administration of nucelot(s)ide analogs is recommended if serum HBV DNA levels increase during DAA treatment for chronic hepatitis C (http://www.jsh.or.jp/medical/guidelines/jsh_guidlines/hepatitis_c). Thus, it is necessary for us to closely monitor the serum HBV DNA level during and after DAA treatment. In this case, the patient received nucleotide analog. Thereby, serum HBV-DNA level gradually decreased, and liver enzymes improved.

We herein reported a rare case of HBV reactivation from resolved infection status (HBsAg-, anti-HBs-negative, anti-HBc-positive) that developed after DAA treatment in a patient with chronic hepatitis C. This case highlights that reactivation of resolved HBV infection remains a risk following eradication of HCV, suggesting that HCV infection may play a role in suppressing HBV replication.

## Acknowledgments

We thank Gillian Campbell, PhD, from Edanz Group (www.edanzediting.com/ac) for editing a draft of this manuscript.

## Author contributions

**Data curation:** Akio Miyasaka, Yuichi Yoshida, Akiko Suzuki.

**Formal analysis:** Tomoyuki Masuda, Hiroaki Okamoto.

**Supervision:** Hiroaki Okamoto, Yasuhiro Takikawa.

**Writing – original draft:** Akio Miyasaka.

**Writing – review & editing:** Akio Miyasaka.

## Abbreviations

Abbreviations: anti-HBc = antibody against HBV core, anti-HBs = antibody against HBsAg, DAA = direct-acting antiviral, HBV = hepatitis B virus, HBsAg = hepatitis B surface antigen, HCV = hepatitis C virus, RBV = ribavirin, SOF = sofosbuvir.
